# Effect of iron chelation on anti-pseudomonal activity of doxycycline

**DOI:** 10.1016/j.ijantimicag.2021.106438

**Published:** 2021-12

**Authors:** ME Faure, A Cilibrizzi, V Abbate, KD Bruce, RC Hider

**Affiliations:** a School of Cancer and Pharmaceutical Sciences, King's College London, London, SE1 9NH, United Kingdom.; b Department of Analytical, Environmental and Forensic Science, King's College London, London SE1 9NH, United Kingdom

**Keywords:** Antimicrobial resistance, doxycycline, iron chelation, Synergy, *P. aeruginosa*

## Abstract

**Background:**

Increasing resistance of microorganisms to antimicrobial agents is a growing concern and there is a lack of novel agents. This has stimulated the exploration of novel strategies for treatment of infection.

**Objective:**

To investigate synergistic interactions between five tetracyclines and tobramycin with an iron chelator (CP762) against two reference strains and nine clinical isolates of *Pseudomonas aeruginosa* from cystic fibrosis patients.

**Method:**

Microdilution assays for minimal inhibitory concentration determination and checkerboard assays were used to assess synergy between antibiotics and CP762. Given the iron-binding capacity of tetracyclines, the binding of iron with doxycycline was investigated using Job's plot methodology. Synergy between the iron-bound form of doxycycline and CP762 was compared with that of unbound doxycycline and CP762. Enhancement of doxycycline anti-biofilm activity was also assessed.

**Results:**

There was synergy between CP762 and all tetracyclines, except minocycline, against the reference strains but that against clinical isolates was variable. Synergy was not demonstrated for tobramycin against any of the strains tested. This led to the hypothesis that iron chelation preserves the binding of tetracyclines to the bacterial ribosome. Susceptibility to iron-bound doxycycline was decreased by two- to four-fold and synergistic interactions with the iron chelator were consistently more intense with iron-bound doxycycline than with doxycycline alone. The doxycycline–iron chelator combination also significantly reduced cell viability in established biofilms.

**Conclusion:**

The data in this study provide evidence that iron chelation enhances the anti-pseudomonal activity of tetracyclines, specifically doxycycline.

## Highlights

• High affinity iron chelation enhances the antibacterial activity of tetracyclines.
• High affinity iron chelation synergises with doxycycline against *P. aeruginosa.*
• Doxycycline chelates iron and loses antibacterial activity.
• Iron chelation re-establishes the susceptibility of iron bound doxycycline.
• Iron chelation enhances doxycycline activity in a biofilm setting.

## 1 Introduction

Antimicrobial resistance of opportunistic pathogens is an increasing global health concern, and few novel antibiotic agents have entered clinical use since the turn of the century [1]. One such opportunistic pathogen, *Pseudomonas aeruginosa,* causes topical and superficial as well as life-threatening infections in susceptible individuals and is particularly challenging to treat because of its natural and acquired resistance to a wide range of antimicrobials [2]. Individuals with cystic fibrosis (CF) are highly susceptible to respiratory acquisition of *P. aeruginosa* infection, which often progresses to a chronic state and associated morbidity and mortality [2]. Combinations of antibiotics are often used in CF airway infection management, with varying clinical success. This study was conducted to design synergistic combinations for the treatment of a range of pathogens using a chemical protective approach. The concept of antibiotic protection, which is based on the interaction between an antibiotic and another chemical present in the environment, is likely to be independent of the type of pathogen.

Tetracyclines are established chelators [3], [4], [5], with a high affinity for copper, iron and zinc, and lower affinities for magnesium and calcium [3,4,6]. Tetracyclines bind to the 30S bacterial ribosome through a magnesium bridge [7,8]. Abundant metals, such as iron, may interfere with this mechanism by binding to the magnesium binding site. The working hypothesis for the current study is that CP762 sequesters iron thereby minimising iron-binding to other ligands, e.g., tetracycline. This will promote complexation with lower affinity ions, such as magnesium, required for binding to the bacterial ribosome. Tetracyclines are not antibiotics of choice for treating *P. aeruginosa* infections; however, *P. aeruginosa* is a relevant study model of iron starvation using diverse iron-acquisition systems and the production of high-affinity siderophores, such as the pigment pyoverdine, which is one of the most potent microbial siderophores [9]. Furthermore, *P. aeruginosa* is very difficult to treat and is therefore an important target for the development of innovative therapeutic strategies. Several studies have indicated the benefit of using iron chelation in conjunction with tetracyclines, including against *P. aeruginosa* [6,10,11], *Burkholderia cepacia* [11], *Acinetobacter baumannii* [6], *Plasmodium falciparum* [12], and *Candida albicans* [13]. Other iron-complexing antibiotics, such as the quinolone ciprofloxacin, may also benefit from a similar chelator-based protective mechanism [14].

**Figure 1:**
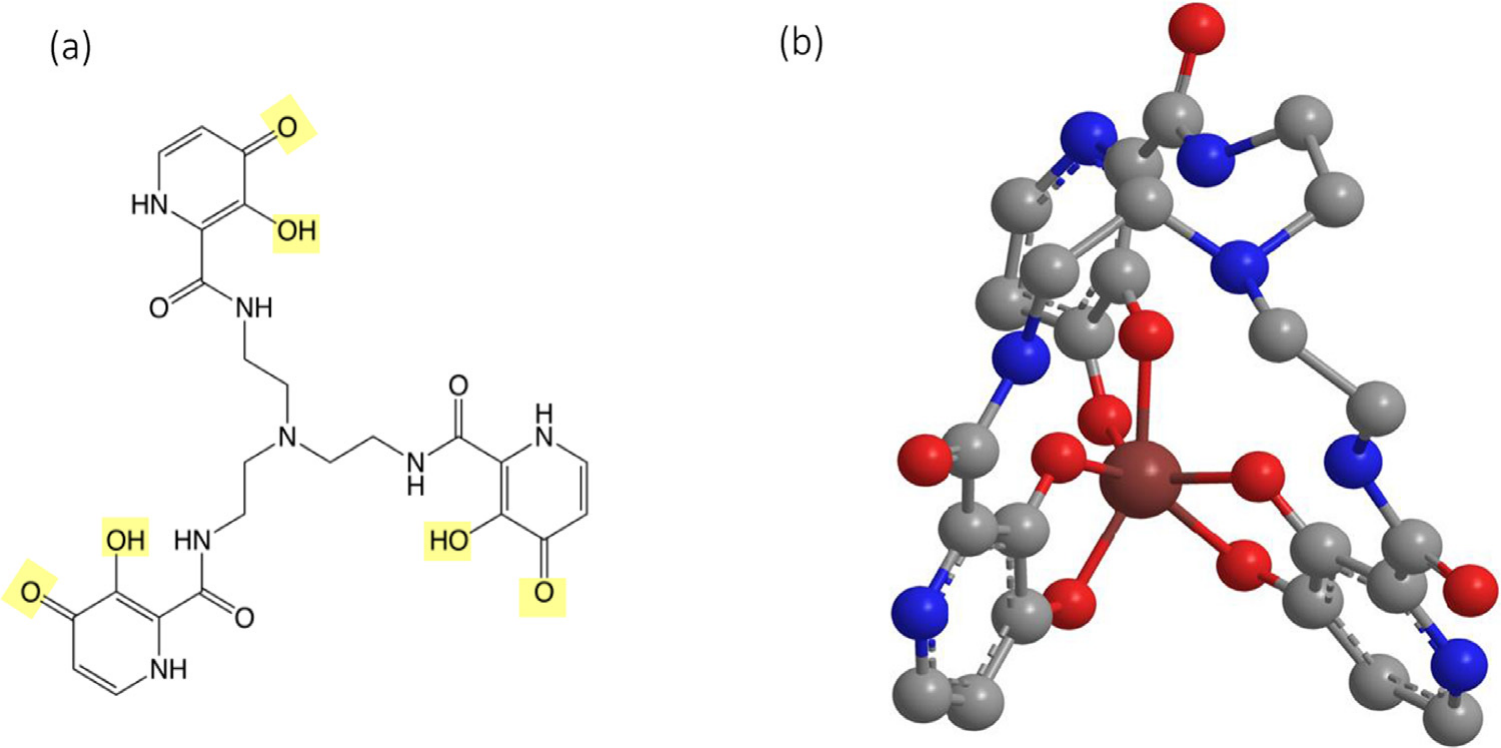
Iron chelator CP762 and iron complex CP762 (a) is a tri-hydroxypyridinone ligand; (b) iron bound to CP762 in a hexadentate fashion. Hydroxypyridinone ligands have the characteristic of other binding groups found in nature, such as enterobactin or deferoxamine, without oxidation risk or degradation (for a detailed review on hydroxypyridinones, see [22]). Metal coordination is through the two vicinal oxygen atoms (highlighted in yellow, a) present on three aromatic rings providing an ideal configuration to ensure high selectivity for iron and pH stability of both ligand and complex [22,23].

In principle, there are several iron chelators (e.g., natural siderophores, chelators in clinical use) that could be used to influence this interaction. The problem with siderophores is the possibility that the corresponding iron complex will supply iron to the microorganism and impact the influence of the siderophores. Previous studies have used deferoxamine in conjunction with antibiotics [15,16] but a range of bacterial species have developed acquisition systems for deferoxamine [17,18]. The most widely used, clinically useful, non-siderophore iron chelators are hydroxypyridinone, deferiprone, and the phenol-containing deferasirox. The latter two are not hexadentate and have lower affinities for iron, hence are not ideal for scavenging iron at low concentrations. Furthermore, deferasirox is associated with numerous side effects and is unsuitable for antibacterial therapies [19]. In contrast, many of the hydroxypyridinone family have proved to be clinically useful [20,21], with bidentate and hexadentate hydroxypyridinones having multiple applications [21]. The hexadentate chelator, CP762 has very high affinity and selectivity for iron (Figure 1) [22,23] and does not utilise many of the bacterial iron-siderophore receptors [23], which means it is unlikely to donate iron to pathogenic microorganisms. In addition, CP762, through iron chelation, has shown antibacterial activity against Gram-negative and Gram-positive pathogens [24].

In this study, the combination of a range of tetracyclines with CP762 in inhibiting *P. aeruginosa* growth and in synergising with antibiotics that have iron-chelating properties was explored. This study assessed the impact of removing iron, an essential growth factor for *P. aeruginosa*, through iron chelation with CP762 alone and in combination with the protein synthesis-inhibiting antibiotics, tobramycin and tetracycline. As tetracyclines are established iron chelators, the impact of iron complexation on their antibacterial activity was also investigated.

## 2 Material and methods

### 2.1 Strains and growth conditions

Reference strain *P. aeruginosa* PAO1 (DSM 22644) was purchased from the German National Culture Collection (Braunschweig, Germany) and PA14 was kindly provided by Dr Martin Welch (Cambridge University). The panel of nine CF clinical isolates comprised strain RP73, a characterised multidrug-resistant CF strain from Dr Alessandra Bragonzi (Infection and Cystic Fibrosis Unit, San Raffaele Scientific Institute, Milan, Italy) [25], and eight isolates from respiratory samples of patients with chronic or acute infection from Professor Jane Davies (Imperial College London). The strains were examined for pigment production on King's A and King's B agar (Sigma-Aldrich; Gillingham, UK). Pyocyanin-positive isolates were characterised by a blue/green colour on King's A agar, and the production of pyoverdine by a yellow-green colour and confirmed by fluorescence under UV light on King's B agar. Colony morphotype was examined on non-selective Luria-Bertani (LB) agar and recorded according to previously described colony type [26]. Tobramycin resistance was determined in cation-adjusted Mueller Hinton broth with 4 mg/L defined as the breakpoint according to EUCAST 2016 guidelines.

Susceptibility assays and routine cultures were performed using LB Lennox media (Becton-Dickinson, Wokingham, UK). All antibiotics were purchased from Sigma-Aldrich; stock solutions were prepared according to the manufacturer's instructions, filter sterilised (0.22 µm) and stored at -20 °C. CP762 (Figure 1) was synthesised and characterised as previously described [22]. Stock solution concentrations were adjusted using molar absorbance coefficients at 330 nm (E_330_=28 624 L mol^−1^ cm^−2^).

### 2.2 Iron-free media and maintaining constant iron levels

All glassware was acid-washed three times prior to autoclaving. Ultra-pure water was used for stock solutions, media and assays. To ensure constant and low iron levels in assays, an aliquot of each batch of reagents was diluted 5 to 10 times in 8% nitric acid (Fisher Scientific, Loughborough, UK) and analysed by inductively coupled plasma mass spectrometry (The London Metallomics Facility, KCL). Iron in water and saline was not detectable (below baseline) and LB contained an average of 5.4 ± 0.16 mM (n=8) iron.

### 2.3 Doxycycline-iron complex stoichiometry and ligation chemistry

Ligation of doxycycline at 0.1 mM with iron (Iron ICP standard in 2% HNO_3,_ Fisher Scientific) was carried out in ultra-pure water and diluted in MOPS (3-(N-morpholino)propane sulfonic acid (Fisher Scientific) at 0.2 M, pH 7.0. To investigate the stoichiometry of iron binding with doxycycline, 10 different molar fractions of the latter, ranging from 0 to 200 µM, were incubated with different ratios of iron, and full-range UV and visible spectra were obtained in MOPS pH 7.0. The maximal absorption difference between ligand (doxycycline) and complex (doxycycline-iron) was observed at 425 nm. Absorbance of each molar fraction at 425 nm was used to produce a Job's plot [27]. For susceptibility testing using the doxycycline-iron complex, the ligation solution (1:1 ratio) was kept at room temperature for 1 h, diluted 10 times with PBS pH 7.4, and filter-sterilised before storage at -20 °C. The pH of growth medium supplemented with doxycycline or complex remained neutral. The minimum inhibitory concentration (MIC) assay was then carried out as described above using doxycycline or doxycycline-iron complex.

### 2.4 Antibacterial susceptibility and synergy testing

MICs were determined in flat-well plates (Greiner BioOne, Stonehouse, UK) by microdilution assay with minor modifications [28]. Briefly, two-fold serial dilutions of each antimicrobial agent were made in sterile ultra-pure water and double-strength LB added to give a bacterial suspension of around 5x10^5^ CFU/mL. Plates were sealed with a gas-permeable membrane (Greiner) and incubated without shaking for 18-22 h at 37 °C. MIC was defined as the lowest concentration of antibiotic to inhibit visual bacterial growth after incubation.

For synergy testing, checkerboard assays were performed following the same procedure and a matrix of 7x7 challenge combinations per plate. Fractional inhibitory concentration indices were used to assess synergy and were determined as follows: FICI = FIC_A_ + FIC_B_ where FIC_A_ = MIC_A+B_ / MIC_A_ and FIC_B_ = MIC_B+A_ / MIC_B_. An FICI ≤0.5 denoted synergy [29]. For ease of comparison between antibiotics with different MICs (i.e., doxycycline versus doxycycline-iron complex), concentrations at the synergistic point were normalised using the MIC of each agent and the checkerboard interfaces were plotted as isobolograms.

### 2.5 Biofilm viability assays

The impact of the interaction between doxycycline and iron chelator was assessed on established biofilms of strain PA14 using the minimal biofilm eradication concentration (MBEC) assay with minor modifications [16,30]. One colony of the test strain was inoculated in LB broth and incubated overnight at 37 °C with shaking at 150 rpm. Bacterial cells were washed twice after centrifugation (5000*g* for 10 min), resuspended in sterile 0.9% NaCl, and diluted to approximately 10^5^ CFU/mL in LB broth; 150 µL of this was aliquoted per well and closed with a Nunc-TSP 96 Pin lid (Nunc, Thermo Fisher Scientific) to allow biofilm formation. After 20 h incubation at 37 °C with gentle agitation (120 rpm), biofilms formed on pegs were washed with PBS (200 µL) and placed in a challenge plate. The latter contained serial two-fold dilutions of doxycycline in PBS pH 7.4, with a constant concentration of CP762 at 1xMIC (32 mg/L) or 0.5xMIC (16 mg/L), or PBS alone as the control. After 20 h, biofilm pegs were washed, placed in PBS (200 µL per well) and the plates sonicated in a water bath for 15 min in a stainless-steel insert. Bacterial cell viability in the resuspended biofilm material was assessed by dilution (1:10) and plating of 20 to 50 µL of each on LB agar for CFU enumeration after 24 h at 37 °C.

## 3 Results

### 3.1 Activity of CP762 combined with tobramycin and tetracyclines against reference and clinical strains of *P. aeruginosa*

**Figure 2:**
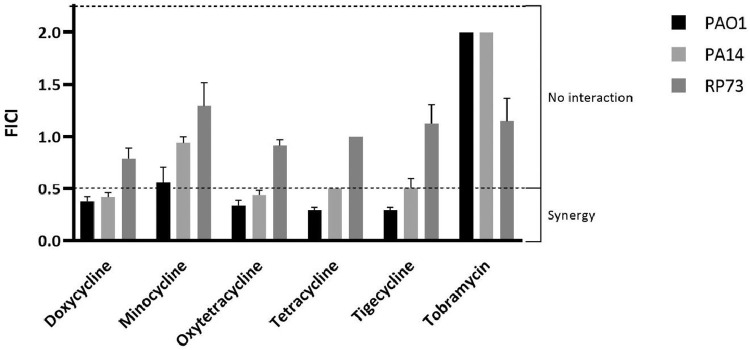
Interaction of CP762 with antibiotics assessed against P. aeruginosa FICIs (fractional inhibitory concentration indices) were determined using the checkerboard assay assessing synergy level between tobramycin, tetracycline antibiotics and iron chelator for P. aeruginosa strains PAO1, PA14 and RP73. Assays were performed three times in duplicate (n=6), bars represent SEM.

CP762 was assessed for synergy with tobramycin and five members of the tetracycline family against the reference strains PAO1 and PA14 and the characterised CF isolate RP73 (Figure 2). Both PAO1 and PA14 produced pyoverdine (Table 1) and exhibited resistance to CP762 alone (32 mg/L or 57 µM). Strain RP73 was negative for pyoverdine (Table 1), which was consistent with the corresponding low MIC values for the chelator (4 mg/L or 7.2 µM). A range of outcomes of synergy tests with CP762 and antibiotics with the reference strains was observed (Figure 2). No combination was clearly synergistic against strain RP73, although doxycycline plus chelator gave a value marginally above (FICI=0.8) the positive synergy cut-off (FICI>0.5) point. Minocycline was found not to be synergistic with CP762 for strains PAO1 and PA14; however, all other tetracyclines displayed synergistic interactions (FICI≤0.5). Tobramycin did not show any synergy with the chelator against any of the strains tested.

**Table 1:** *P. aeruginosa* isolates used in this study The panel of isolates consisted of two reference strains (PAO1, PA14), a characterised CF isolate (RP73) and eight CF isolates from acute or chronic infection.

Strains		Stage of infection	Pigments			Morphotype	Tobramycin resistance
			Pyoverdine		Pyocyanin		
Reference strains	PAO1		+		+	Typical	S
	PA14		+		+	Typical	S
Clinical isolates	RP73		-		+	Dwarf	R
	CF02	First	+		+	Typical	S
	CF08	Chronic	+		+	Typical	S
	CF10	Chronic	+		-	Feather like	S
	CF11	Chronic	-		+	Dwarf	R
	CF13	Chronic	+		+	Typical	S
	CF15	Chronic	+		+	Typical	S
	CF19	Chronic	-		-	Dwarf	R
	CF26	First	+		+	Feather like	S

To investigate whether the observed synergy extended to clinical isolates from CF patients in which these antibiotics would be used, the susceptibility of the CF panel isolates to doxycycline, selected as a representative of the tetracycline group, and tobramycin, both alone and in combination with CP762, was determined; the chelator alone was also tested for inhibitory activity (Tables 2 and 3). The MIC for CP762 alone ranged from 4 to 32 mg/L, with the pyoverdine-deficient strains (RP73, CF11 and CF19) showing the highest susceptibility. All strains except two exhibited a doxycycline MIC of 8 mg/L; the outliers (CF02 and CF10) had an MIC of 32-64 mg/L. Tobramycin MICs ranged from 1 mg/L to 2-4 mg/L for susceptible strains as defined by EUCAST (Table 1); three were resistant, with MICs of 8 to 32 mg/L.

**Table 2:** MICs of doxycycline and CP762 alone and in combination Checkerboard assays were performed in LB against 11 isolates of *P. aeruginosa*. MICs were determined for agent alone or in combination. Data are the mean of 6 replicates, minimal and maximal values between brackets when applicable (i.e., when MICs varied between replicates). The mean FICI were then calculated and used to plot data

Strains		MIC doxycycline (mg/L)			MIC CP762 (mg/L)			Mean FICI
		Alone		In combination	Alone		In combination	
Reference strains	PAO1	8		1.7 (1 - 2)	32		4.7 (2 - 8)	0.38
	PA14	8		2	32		7.3 (4 - 8)	0.42
Clinical isolates	RP73	8		3.0 (2 - 4)	4		1.7 (1-2)	0.79
	CF02	64		8.0 (2 - 16)	16		2	0.24
	CF08	8		2.2 (1 - 4)	32		6.7 (4 - 8)	0.48
	CF10	32		12 (4 - 16)	32		11 (8 - 16)	0.60
	CF11	4		4	6.7 (4 – 8)		6.7 (4 – 8)	2.0
	CF13	8		1.4 (0.5 - 2)	32		5.3 (4 - 8)	0.30
	CF15	8		0.92 (0.5 - 1)	32		2.7 (1 - 4)	0.18
	CF19	8		6.7 (4 - 8)	6.7 (4 – 8)		5.3 (4 - 8)	1.7
	CF26	8		1.3 (1 - 2)	32		6.7 (4 - 8)	0.38

**Table 3:** MICs of tobramycin and CP762 alone and in combination Checkerboard assays were performed in LB against 11 isolates of *P. aeruginosa*. MICs were determined for agent alone or in combination. Data are the mean of 6 replicates, minimal and maximal values between brackets when applicable (i.e., when MICs varied between replicates). The mean FICI were then calculated and used to plot data.

Strains		MIC tobramycin (mg/L)			MIC CP762 (mg/L)			Mean FICI
		Alone		In combination	Alone		In combination	
Reference strains	PAO1	2		2	32		32	2
	PA14	2		2	32		32	2
Clinical isolates	RP73	8		5.0 (2 – 8)	4		2.7 (2 – 4)	1.2
	CF02	1		0.75 (0.5 – 1)	16		12.0 (8 - 16)	1.5
	CF08	2		1.8 (1 – 2)	32		29 (16 – 32)	1.8
	CF10	1		1	32		32	2
	CF11	27 (16 - 32)		24.0 (16 – 32)	6.7 (4 – 8)		6.0 (4 – 8)	1.8
	CF13	2		2	32		32	2
	CF15	2		2	32		32	2
	CF19	32		27 (16 – 32)	6.7 (4 – 8)		5.3 (4 – 8)	1.7
	CF26	2.7 (2 - 4)		3.7 (2 – 4)	32		32	2.5

**Figure 3:**
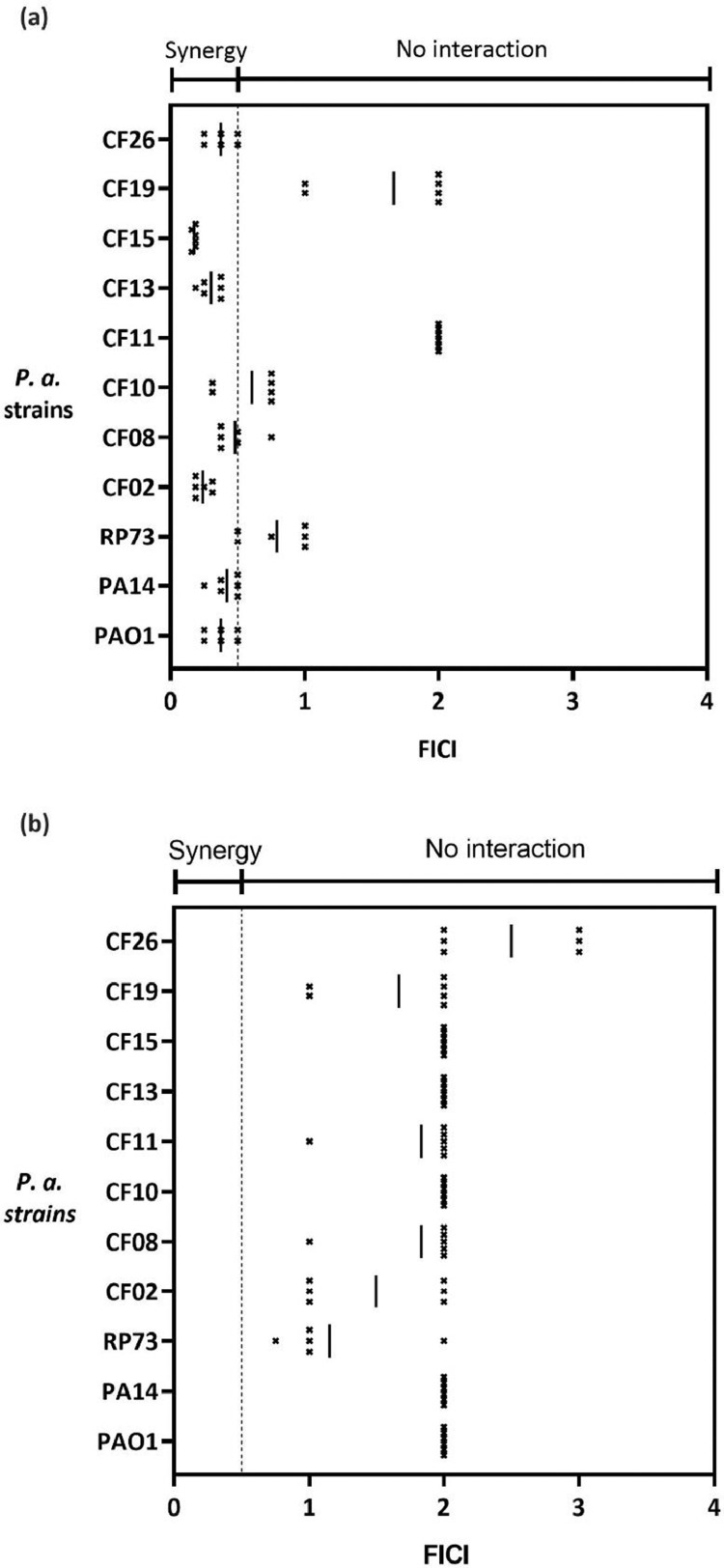
Interaction between CP762 and doxycycline (a) or tobramycin (b) Doxycycline – CP762 (a) and tobramycin – CP762 (b) combinations were tested on 11 strains of P. aeruginosa using the checkerboard assay. Points represent FICI produced by individual checkerboard and bars represent the mean FICI produced by 3 independent experiments in duplicate (n=6).

Fractional inhibition concentration data showed synergy (FICI<0.5) between doxycycline and CP762 (Figure 3a) for seven of the 11 strains tested. Of the CF panel, five showed synergy and the remainder showed additivity or indifference (FICI<2). No synergistic activity between tobramycin and CP762 was found (Figure 3b). Interestingly, all strains positive for synergistic interaction were pyoverdine producers whereas three of the four strains that were 'insensitive’ to doxycycline-CP762 (FICI<0.5) were negative for pyoverdine production. This cluster also displayed resistance to tobramycin (strains RP73, CF11 and CF19), high susceptibility to CP762 and exhibited similar morphotypes (dwarf, Table 1).

### 3.2 Characterisation of iron-doxycycline complexes

**Figure 4:**
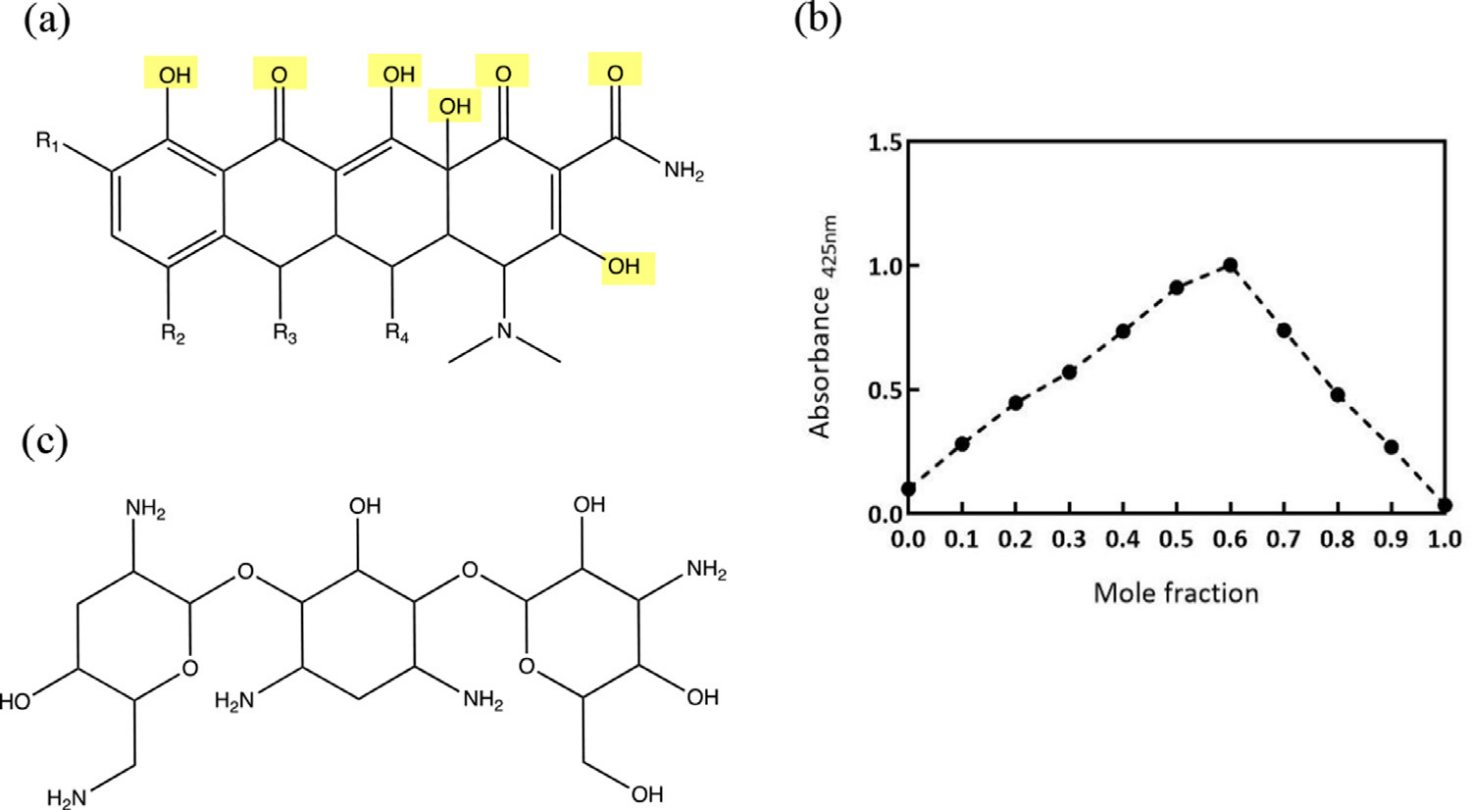
Iron complexation with doxycycline. Stoichiometry of binding of doxycycline and iron determined by UV-Vis absorbance Chemical structures of tetracycline general structure (a, doxycycline R1=R2=R3=H; R4=OH) and tobramycin (c) were examined in relation to their iron-binding potential; possible iron complexation sites are highlighted in yellow. The Job's plot (b) was produced using absorbance at 425 nm of doxycycline and iron at different molar fractions.

The next part of the study was to assess whether doxycycline coordinated iron under the conditions tested and whether this complexation affected its antibacterial activity. Tobramycin was used as an antibiotic control. The chemical structures of both antibiotics in relation to their possible iron-binding sites are shown in Figures 4a and c. Figure 4a shows several potential iron coordination sites (highlighted in yellow). In contrast, Figure 4c shows a complete lack of iron coordination sites. Although tobramycin contains several hydroxyl groups, these are aliphatic and therefore have little affinity for iron. Thus, the stoichiometry of iron binding was investigated for doxycycline only. The Job's plot [27] (Figure 4b) gave a peak corresponding to a molar fraction of 0.6, indicating a stoichiometry of 3:2 (i.e., 3 doxycycline molecules binding 2 atoms of iron). However, based on its structure, doxycycline was considered unlikely to form a 3:2 complex, particularly at low concentrations. This result was interpreted as representing an equimolar mixture of ratio 1:1 (mole fraction 0.5) and ratio 2:1 (mole fraction 0.7). The ratio 1:1 was selected for the remainder of the study because this will be the dominant complex present in solution at the concentrations used in the microbiological assays. All the tetracyclines, including doxycycline (Figure 4a), contain many potential binding sites suitable for chelation (highlighted in yellow). In contrast, tobramycin (Figure 4c) lacks such high-affinity binding sites and this most likely accounts for the lack of synergy presented in Figure 3.

### 3.3 Synergy between CP762 and doxycycline prevents iron-mediated antibiotic deactivation

The enhanced antibacterial effect of doxycycline in the presence of CP762 may be due to a protective effect of the iron chelator, preventing antibiotic deactivation. A comparison of the antibacterial effect of doxycycline with its iron-complexed form showed that susceptibility to doxycycline was moderately decreased by two- to four-fold when bound to iron (MIC increase from 8 to 16-32 mg/L), as shown in Table 4.

**Table 4:** Effect of CP762 on *s*usceptibility to doxycycline and doxycycline-iron complex MICs of doxycycline or doxycycline-iron complex against strains PAO1, PA14 and RP73 were examined for antibiotic used alone or in combination with CP762 at 0.25 or 0.5 x MIC in the checkerboard assay. Corresponding FICI are shown as mean of three independent replicates ± SDs. Assays were carried in LB containing 5.3 µM iron. CP762 concentrations were 8 or 16 mg/L (14 or 28 µM) for PAO1 and PA14 and 2 and 4 mg/L (4 or 7 µM) for RP73.

Strain	MIC (mg/L)							
	Without CP762		CP762 0.25 x MIC		CP762 0.5 x MIC		FICI	
	Doxycycline	Doxycycline-iron complex	Doxycycline	Doxycycline-iron complex	Doxycycline	Doxycycline-iron complex	Doxycycline	Doxycycline-iron complex
PAO1	8	16	2-4	4-8	2-4	4	0.31 ± 0.04	0.25 ± 0.03
PA14	8	32	2	2-4	1-2	1-2	0.38 ± 0.05	0.21 ± 0.03
RP73	8	16	8	8	2-8	4-8	0.92 ± 0.05	0.54 ± 0.05

As expected, in the presence of CP762, antibiotic susceptibility was re-established for strains PAO1 and PA14, with the MIC reduced by a factor two- to 16-fold, highlighting the rescue of susceptibility by the iron chelator. FICI was markedly lower for the doxycycline-iron complex (0.25 and 0.21) compared with doxycycline alone (0.31 and 0.38) for both strains. The effect was weaker for RP73, which was insensitive to the combination (FICI=0.92) when using the iron-free form of doxycycline. However, susceptibility with the doxycycline-iron complex was increased by a factor of two to four, resulting in a much lower FICI (0.54).

**Figure 5:**
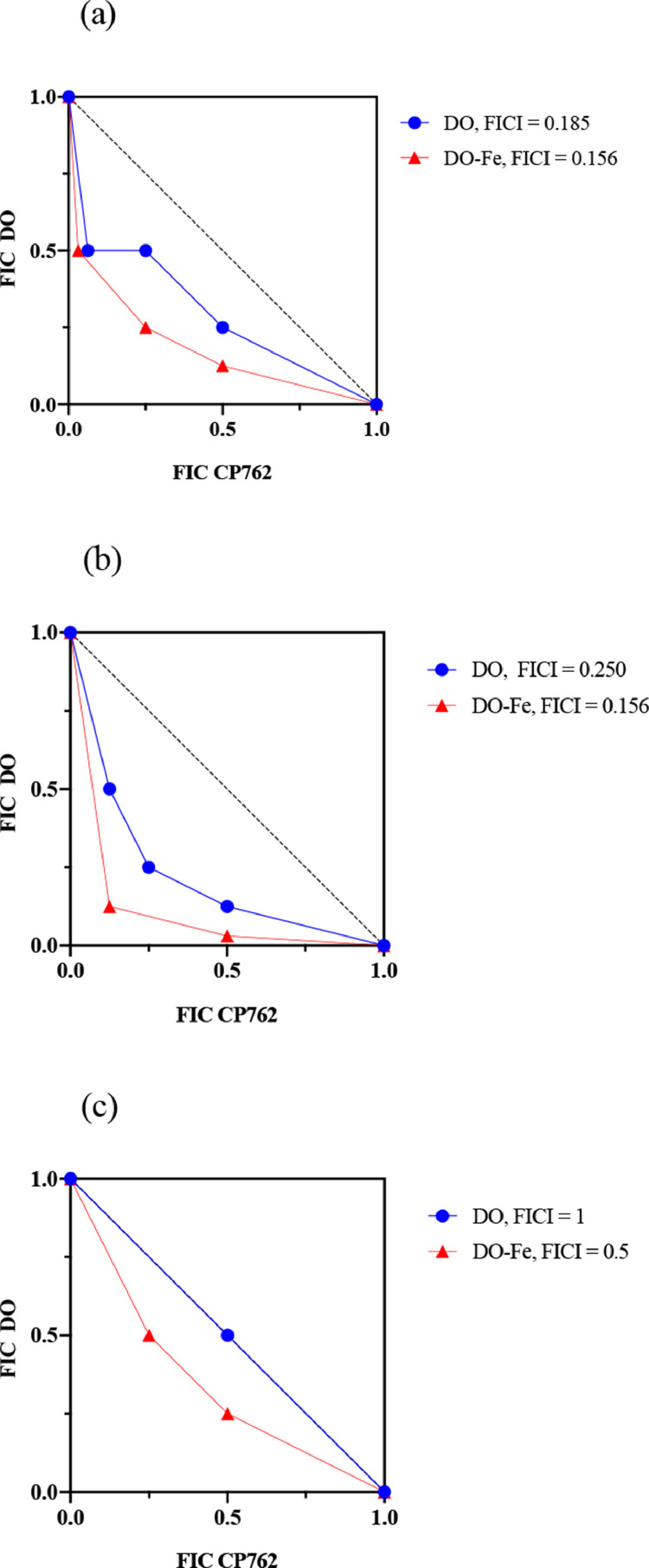
Isobolograms showing synergy between iron chelator and doxycycline or doxycycline-iron complex Checkerboard assay was repeated three times in LB to investigate synergy between doxycycline (DO, circles) or doxycycline-iron complex (DO-Fe, triangles). The figure shows one biological replicate for each of the strain PAO1 (a), PA14 (b) and RP73 (c). As MICs were different for DO (MIC 8 mg/L) and DO-Fe (MIC 32 mg/L), results were normalised (concentration for synergy point / MIC) and plotted as isobolograms; differences in curve shape highlight differences in synergy level. The additivity line (FICI = 1) is represented by the black dotted line.

The effect was investigated further to determine whether the chelator produced a higher degree of synergy with the doxycycline–iron complex compared with doxycycline alone. Figure 5 shows an example of the normalised checkerboard results against the three *P. aeruginosa* strains. As expected, isobologram analysis produced concave isoboles (below additivity line) for both PAO1 and PA14. In the case of RP73, only the doxycycline–iron isobole was under the additivity line. In addition, all isoboles for the doxycycline–iron complex were consistently more concave than that of the antibiotic alone, indicating different levels of synergy.

### 3.4 The iron chelator CP762 increases doxycycline killing effect in a biofilm setting

**Figure 6:**
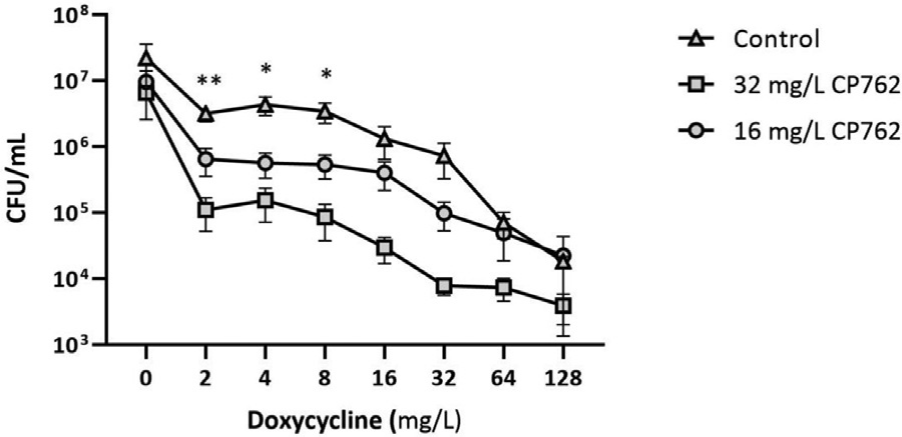
Effect of CP762 on doxycycline bactericidal activity against established biofilms of PA14 Established biofilms grown in untreated LB were exposed to doxycycline alone (triangles) or in the presence of CP762 at a constant concentration of 16 mg/L (circles) or 32 mg/L (squares). Results are the mean of three independent replicates, bars show SEM. *P < 0.05 and **P < 0.01 non-treated control vs. CP762 at 16 or 32 mg/L (unpaired student t-test).

As *P. aeruginosa* persistence is often associated with biofilm, it was logical to investigate the in vitro activity of combined doxycycline and iron chelator against established biofilms. MBEC assays proved unhelpful as doxycycline, even at the highest doses, failed to fully eradicate the established biofilms. This resulted in visible bacterial growth in all wells of the inhibitor-free/recovery media and absence of MBEC values. The MBEC assay was thus set up to assess cell viability within the biofilm structure rather than absolute killing/biofilm removal (Figure 6). Doxycycline greatly reduced the number of viable cells in the biofilm of strain PA14 in a dose-dependent manner. Cell enumeration showed a 3 log_10_ reduction in viable count between the non-treated control (2.3 x 10^7^ CFU/mL) and biofilms treated with the highest dose of doxycycline (128 mg/L, 1.8 x10^4^ CFU/mL). The addition of CP762 at 16 mg/L or 32 mg/L to the antibiotic-treated control did not significantly alter cell viable counts compared with the untreated control. Nonetheless, doxycycline (2 to 32 mg/L) exhibited an increased bactericidal effect in the presence of CP762 at 16 mg/L and this was enhanced at 32 mg/L. Significant differences in cell counts were observed for doxycycline at 2 (*P*<0.01), 4 and 8 mg/L (*P*<0.05) with both concentrations of CP762.

## 4 Discussion

A variablegree of synergistic interaction between CP762 and four of five tetracycline antibiotics tested was demonstrated for two reference strains of *P. aeruginosa* (Figure 2), with doxycycline exhibiting the highest inhibitory effect in combination with the chelator. This effect was corroborated in further testing of nine CF isolates. *P. aeruginosa* acquires iron through multiple mechanisms, including direct acquisition from haem and host proteins through siderophore-driven uptake, and production of phenazine compounds that reduce transferrin-bound iron [31,32]. A cluster of three strains that were deficient for pyoverdine production and ‘insensitive’ to the combination (0.5<FICI<2) was identified. These CF isolates were highly susceptible to CP762, as shown by the lowest MICs obtained (4 to 8 mg/L) corresponding to 3.7 to 7.2 µM of CP762. This concentration was equivalent to the total molar concentration of iron in LB broth (5.4 µM). Thus, these isolates grew in conditions where iron and CP762 were in molar equivalent, in contrast to the rest of the group, which grew when CP762 was in large molar excess. These isolates have likely developed an alternative to pyoverdine production and may be considered as ‘pyoverdine cheaters’, a phenotype of clinical relevance in CF [33] that does not produce siderophores with a similar affinity for iron to CP762, thus explaining the low MICs.

Nevertheless, because of their high susceptibility to iron chelation, they might not be suitable for checkerboard analysis as a technical limitation of the assay may mask the interaction. Synergism is declared in the checkerboard assay when the MICs of combined agents are decreased >4-fold. For this group of isolates, however, a 4-fold decrease in MIC corresponds to 1.75-3.5 µM CP762, which is far below the total concentration of iron in LB broth. Thus, a complete inhibition of bacterial growth below this threshold is highly unlikely and, mathematically, an FICI <0.5 could not be produced. Thus, the response of these isolates to the combination of iron chelation and antibiotic should be carefully interpreted and perhaps only used as a rationale for additional investigations.

Tobramycin, another antibiotic that inhibits protein synthesis, was shown to bind the bacterial ribosome via water-bridged hydrogen bonds [34]. To the best of our understanding, there is no evidence of iron interactions with tobramycin binding to the bacterial ribosome, and the lack of synergy and antagonism in this study is consistent with this. Only one previous study has investigated whether tobramycin and a tetracycline antibiotic (tigecycline) susceptibility could be enhanced in the presence of iron chelators [10]. In this earlier study, there was evidence of modulation of tobramycin resistance according to iron availability (this was dependent on the iron source: haem versus inorganic iron), which was suggestive of an interaction between iron metabolism and tobramycin activity. Tigecycline resistance, however, appeared to operate through another mechanism in that iron supplemented as haem did not affect its activity whereas inorganic iron enhanced resistance to this agent. The study authors speculated that this was indicative of a direct interaction of iron with antibiotic. The current study demonstrated that the synergy between CP762 and the antibiotics was stronger when using the iron-doxycycline form compared with the free ligand. This finding supports the hypothesis that synergy between iron chelator and doxycycline is, in part, the result of a physical protective mechanism preventing iron binding to the antibiotic.

The chemistry of complex formation between metals and tetracyclines is influenced by solvent, pH, and other conditions, as reflected in the conflicting reports on tetracycline-metal stoichiometry [3], [4], [5]. Only two studies have focussed on doxycycline-iron stoichiometry, both of which reported the formation of 2:1 complexes [35,36]. In the current study, both 1:1 and 2:1 ratios were clearly identified to dominate under physiological conditions. Furthermore, the dilute solutions typically used in microbiological studies are likely to lead to the 1:1 ratio being the most abundant. In addition, using equimolar concentrations of doxycycline and iron in preference to the 2:1 ratio would minimise the possibility of a false-positive synergistic signal.

In this study, it was considered important to use characterised strains and recent isolates from active infections, given the likelihood that these would be biofilm producers and have multiple means of antibiotic resistance. Combining an antibiotic that has iron-binding sites with an iron chelator is promising, particularly for infections associated with biofilms. *P. aeruginosa* causes persistent infections through the formation of biofilms, which mediate adherence to various biomaterials [37], [38]. Iron is essential for all life forms and its importance in relation to biofilm formation and pathogenesis is well-documented [31,32,38]. Biofilms are mainly composed of extracellular polymeric substances that are bound together through ionic interactions and were previously shown to prevent antibiotic penetration through altered diffusion and sequestration [39]. Therefore, it was important to assess whether the enhancement of doxycycline activity through iron chelation was translated in such structures. CP762, in combination with doxycycline, markedly reduced the viability of the test strain (PA14) in biofilms, even at low antibiotic concentrations. Indeed, the greatest reduction of viability was evident with 2-16 mg/L doxycycline and 32 mg/L CP762. The CP762-doxycycline combination had a clear anti-biofilm effect, and the bactericidal action of doxycycline was significantly enhanced by the chelator.

Although the focus of this study was on antibiotic impact, the difference in sputum iron ranges between healthy controls (0–15 µM) and stable CF patients (8-118 µM) needs to be considered [40]. By comparison, synergistic activity of doxycycline with the chelator was evident in LB broth containing an average of 5.3 µM iron. This finding indicates a potential use for compounds such as CP762 as therapeutic adjuncts and shows that further studies are warranted. CP762 is an iron-scavenging compound and is expected to have various levels of activity depending on the infection site or testing environment and the level of iron. Thus, a major limitation of this study is linked to the checkerboard assay because iron, a direct inhibitor of CP762, was present at a constant level despite the gradient of antibiotic and chelator. In retrospect, other assays, such as those assessing the effect of drugs at fixed sub-inhibitory concentrations (time-kill assay) on the number of bacterial cells, could be relevant to assess the adjuvant properties of CP762.

In conclusion, iron chelation through CP762 was found to enhance the activity of five antibiotics belonging to the tetracycline family against *P. aeruginosa*. There was synergy between doxycycline and CP762 for most isolates that produced pyoverdine. Using a novel approach, this study showed different levels of synergy using a pre-made iron-doxycycline complex, highlighting iron-mediated inactivation of antibiotics. The findings indicate that CP762 has potential for use as an adjunct to doxycycline through sequestration of iron, thereby conferring synergistic activity to an antibiotic that would not normally be considered a therapeutic option against *P. aeruginosa*.

## Funding

This work has been supported by internal funding (Institute of Pharmaceutical Science, KCL).

## Ethical Approval

Not required

## Author contribution

MF, AC, VA, KB and RH designed the study. MF and AC performed the experiments and analysed the data. MF and RH wrote the manuscript. All authors approved the final version of the manuscript.

## Declarations of Competing Interests

The authors have no conflict of interests to declare
